# REM Sleep Behavior Disorder as a Key Predictor of Cognitive Decline: Insights from a Narrative Review

**DOI:** 10.1055/s-0046-1819595

**Published:** 2026-05-19

**Authors:** Faezeh Khorshidian

**Affiliations:** 1 Department of Psychiatry, School of Medicine, Tehran University of Medical Sciences, Tehran, Iran

**Keywords:** parasomnias, Parkinson disease, dementia with Lewy bodies, neurodegenerative diseases, alpha-synuclein, polysomnography

## Abstract

**Introduction:**

Rapid eye movement (REM) sleep behavior disorder (RBD) is a parasomnia. Mounting evidence positions RBD as a critical prodromal indicator of synucleinopathies—particularly Parkinson's disease (PD), dementia with Lewy bodies (DLB), and multiple system atrophy (MSA)—with a significant link to progressive cognitive decline.

**Objective:**

To explore the underlying pathophysiological mechanisms connecting RBD with cognitive impairment, to delineate the neuropsychological profile of the affected individuals, and to critically evaluate the current diagnostic tools and therapeutic challenges.

**Materials and Methods:**

We synthesized data from clinical cohort studies, structural and functional neuroimaging modalities (magnetic resonance imaging [MRI], fluorodeoxyglucose positron-emission tomography [FDG-PET], and single-photon emission computed tomography [SPECT]), molecular biomarkers—including alpha-synuclein seeding assays—and genetic studies focusing on mutations such as *glucocerebrosidase, β, acid* ( *GBA* ) and *synuclein alpha* ( *SNCA* ).

**Results:**

Individuals with idiopathic RBD (iRBD) frequently exhibit early deficits in executive function, attention, visuospatial abilities, and episodic memory—often years before motor symptoms manifest. Pathologically, the spread of alpha-synuclein aggregates from REM sleep-regulating nuclei in the brainstem to limbic and neocortical areas mirrors the trajectory of cognitive decline. Cholinergic-system degeneration, particularly in the pedunculopontine nucleus and basal forebrain, further contributes to the neurocognitive symptoms. Neuroimaging consistently reveals frontal and temporal cortical thinning, along with posterior hypometabolism characteristic of synucleinopathies. Biomarker analysis shows altered cerebrospinal fluid (CSF) amyloid-beta and -tau levels, as well as positive alpha-synuclein real-time quaking-induced conversion (RT-QuIC) assay results. Genetic predispositions strongly influence the risk and rate of progression.

**Conclusion:**

A powerful early biomarker for cognitive decline associated with synucleinopathies, the early identification of RDB through comprehensive neuropsychological profiling, advanced imaging techniques, and molecular diagnostic methods is essential for risk stratification and monitoring. Although disease-modifying therapies remain elusive, a deeper understanding of shared pathogenic pathways offers promising avenues for targeted neuroprotective strategies.

## Introduction

Rapid eye movement (REM) sleep behavior disorder (RBD) is a parasomnia characterized by the loss of physiological muscle atonia during REM sleep, resulting in complex motor behaviors that often mirror the content of vivid dreams. 1 2 Once considered a rare and isolated sleep disorder, RBD has garnered substantial attention in recent years due to its strong association with neurodegenerative conditions, particularly synucleinopathies such as Parkinson's disease (PD), dementia with Lewy bodies (DLB), and multiple system atrophy (MSA). 3 4

An increasingly-recognized dimension of RBD is its relationship with cognitive impairment. Numerous studies 3 4 5 6 have demonstrated that individuals with idiopathic RBD (iRBD)—those without an overt neurological diagnosis at the time of presentation—are at markedly-increased risk of developing mild cognitive impairment (MCI) and subsequent dementia. Notably, cognitive deficits may precede the onset of the classic motor symptoms of PD and related disorders by several years, positioning RBD as a critical prodromal marker within the continuum of neurodegeneration. 5 6 7

Understanding the association between RBD and cognitive dysfunction is essential due to its diagnostic and prognostic implications. 5 7 8 Neurobiological investigations 7 8 9 suggest that the underlying pathology of RBD extends beyond the brainstem regions regulating REM sleep atonia, affecting widespread cortical and subcortical areas involved in cognition. This overlap implicates shared pathophysiological mechanisms, such as alpha-synuclein deposition and cholinergic dysfunction, in REM-related motor disinhibition and cognitive decline. 8 9 10

The current narrative review aims to synthesize current evidence linking RBD to cognitive impairment, with an emphasis on clinical characteristics, neuroimaging findings, and the potential mechanisms underlying this association. 8 9 We also highlight key challenges in early diagnosis and discuss implications for therapeutic intervention and disease monitoring. 9 10 11

## Materials and Methods

### Search Strategy and Study Selection

We conducted a systematic literature search on the PubMed, Scopus, Web of Science, and PsycINFO databases, covering publications from inception to April 2025. The search terms included Medical Subject Headings (MeSH) and free-text keywords such as *rapid eye movement sleep behavior disorder* , *RBD* , *cognitive impairment* , *mild cognitive impairment* , *dementia* , *synucleinopathy* , *neuroimaging* , and *biomarkers* . Boolean operators (AND, OR) were applied to maximize sensitivity and specificity. The reference lists of key articles and recent reviews were manually screened to identify additional relevant studies.

**Fig. 1:**
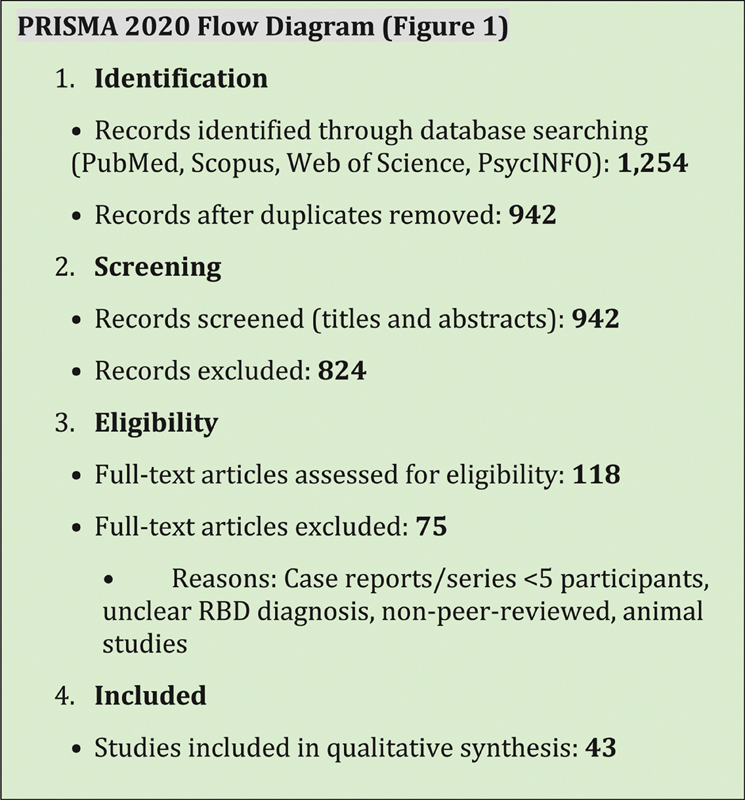
Flowchart according to the Preferred Reporting Items for Systematic Reviews and Meta-Analyses (PRISMA) 2020 statement.

The initial search yielded 1,254 articles. After removing duplicates (n = 312), 942 titles and abstracts were screened, resulting in 118 full-text articles assessed for eligibility. Ultimately, 43 studies met the inclusion criteria and were included in the qualitative synthesis. A Preferred Reporting Items for Systematic Reviews and Meta-Analyses (PRISMA)-style flow diagram is provided in Figure 1 .

### Inclusion and Exclusion Criteria

Eligible studies included:

- Original research articles and meta-analyses examining cognitive function or neurodegenerative outcomes in idiopathic or secondary RBD.
- Clinical studies of adults diagnosed with RBD by polysomnography according to established criteria.
- Investigations reporting neuropsychological assessments, neuroimaging findings, molecular biomarkers, or genetic data related to cognitive impairment.
- Articles published in English.

Systematic reviews were referenced for contextual background but were not included for study-level data extraction.

Exclusion criteria:

- Case reports or series with fewer than 5 participants.
- Studies lacking clear RBD diagnostic confirmation.
- Non-peer-reviewed articles, conference abstracts without full text, and animal studies.

### Data Extraction and Quality Assessment

Data were extracted by two independent researchers, including study design, sample size, RBD diagnostic criteria, cognitive domains assessed, neuroimaging modalities and findings, biomarkers evaluated, and outcomes related to cognitive impairment or neurodegenerative progression.

The methodological quality of the included studies was appraised using standardized tools: the Newcastle-Ottawa Scale (NOS) for observational studies and the Assessment of Multiple Systematic Reviews 2 (AMSTAR-2) checklist for meta-analyses. The quality assessment informed the interpretation of evidence strength and identified gaps in the literature.

### Data Synthesis

Given the narrative nature of the present review and the heterogeneity in methodologies, a qualitative synthesis approach was employed. The findings were organized thematically around clinical cognitive profiles, neurobiological mechanisms, diagnostic strategies and biomarkers, and therapeutic challenges. Discrepancies between researchers were resolved through consensus discussion.

## Pathophysiology Linking RBD and Cognitive Impairment

Rapid eye movement sleep behavior disorder is closely associated with underlying synuclein pathology. The pathological aggregation of alpha-synuclein in brainstem nuclei responsible for REM sleep atonia, such as the sublaterodorsal nucleus and magnocellular reticular formation, also extends to cortical and subcortical regions implicated in cognitive function. 11 12 This widespread neurodegeneration provides a plausible substrate for the cognitive deficits observed in RBD patients. 11 12 13 14

Cholinergic dysfunction appears to play a critical role. Damage to cholinergic nuclei, particularly the pedunculopontine tegmental nucleus (PPT) and the basal forebrain, has been implicated in REM sleep dysregulation and cognitive deterioration. 12 13 14 Additionally, neurodegeneration affecting frontostriatal circuits and limbic structures may further exacerbate executive dysfunction, memory impairment, and emotional regulation deficits in RBD. 13 14 15

## Clinical Features of RBD and Cognitive Impairment

Individuals with iRBD often exhibit subtle but measurable cognitive deficits. These commonly include impairments in attention, executive function, visuospatial processing, and memory. Importantly, these deficits may be detectable years before the development of Parkinsonism or dementia. 15 16 17

Longitudinal cohort studies 16 18 19 have demonstrated that a significant proportion of iRBD patients eventually progress to MCI or meet criteria for a neurodegenerative dementia, particularly DLB or PD dementia (PDD). The progression from iRBD to cognitive impairment is therefore a critical window for early intervention and monitoring.

## Diagnostic Strategies and Biomarkers

Early identification of cognitive impairment in patients with RBD is essential for prognosis and potential therapeutic interventions. Diagnosis typically begins with a thorough clinical assessment, including detailed sleep history and neuropsychological testing. Polysomnography remains the gold standard to confirm RBD, demonstrating REM sleep without atonia (RSWA) and associated dream enactment behaviors according to the latest criteria of the International Classification of Sleep Disorders, 3rd Edition (ICSD-3). 1

➢ **Neuropsychological Assessment**
Comprehensive cognitive testing is critical in identifying subtle deficits in iRBD patients. Standardized assessments often reveal impairments in executive function, working memory, attention, and visuospatial skills—domains commonly affected in synucleinopathies. 20 21 22 Longitudinal monitoring can help track the progression from MCI to more severe neurodegenerative states. 21 22 23
➢ **Neuroimaging Biomarkers**
Structural and functional imaging studies provide further insight into the neural correlates of cognitive decline in RBD. 21 23 24 Magnetic resonance imaging (MRI) studies have demonstrated cortical thinning in the frontal and parietal lobes and hippocampal atrophy in patients with iRBD and cognitive impairment. Functional imaging, including fluorodeoxyglucose positron-emission tomography (FDG-PET) and single-photon emission computed tomography (SPECT), often reveals hypometabolism or reduced perfusion in posterior cortical regions, resembling early changes seen in DLB. 20 21 22 23 24
➢ **Molecular and Fluid Biomarkers**
Emerging research has identified potential molecular biomarkers linking RBD and neurodegeneration. 25 26 27 Cerebrospinal fluid (CSF) analyses may show decreased levels of amyloid-beta and increased total or phosphorylated tau in patients progressing to DLB. Alpha-synuclein seeding assays (such as real-time quaking-induced conversion, RT-QuIC) have shown promise in detecting misfolded synuclein aggregates in CSF or peripheral tissues (such as skin or submandibular gland biopsies), offering diagnostic value before motor symptoms emerge. 25 28
➢ **Genetic Factors**
Genetic variants associated with synucleinopathies may also confer risk in RBD. For instance, mutations or polymorphisms in the *glucocerebrosidase, β, acid* ( *GBA* ), *synuclein alpha* ( *SNCA* ), and *leucine-rich repeat kinase 2* ( *LRRK2* ) genes have been implicated in PD and RBD. While routine genetic testing is not yet standard practice, these associations highlight shared pathophysiology and potential targets for risk stratification ( Table 1 ). 21 23 24

**Table 1:** Comprehensive overview of clinical, neurobiological, imaging, and biomarker features in RBD-associated cognitive impairment

Domain	Feature	Description/Findings	Clinical relevance
**Clinical profile**	Cognitive domains affected	Attention, executive function, visuospatial ability, memory	Early cognitive decline often predates motor symptoms
	Progression rate	Variable; some progress rapidly to dementia, others remain stable	Prognostic importance; guides monitoring and intervention
**Neurobiological basis**	Alpha-synuclein pathology	Aggregation in brainstem, limbic, cortical regions	Shared pathophysiology between RBD and neurodegenerative diseases
	Cholinergic dysfunction	Damage to the pedunculopontine nucleus and basal forebrain	Linked to REM atonia loss and cognitive deficits
	Frontostriatal and limbic circuit involvement	Affects executive functions, memory, and emotional regulation	Explains multifaceted cognitive profile
**Neuroimaging findings**	MRI structural changes	Cortical thinning in the frontal, parietal, temporal lobes; hippocampal atrophy	Correlates with executive and memory impairment
	Functional imaging (FDG-PET/SPECT)	Hypometabolism/reduced perfusion in the posterior cortex	Visuospatial and attentional deficits
	DTI findings	Reduced fractional anisotropy in frontostriatal pathways	Reflects disrupted connectivity linked to cognition
**Biomarkers**	CSF markers	Decreased amyloid-beta, increased total/phosphorylated tau, positive alpha-synuclein seeding assays (RT-QuIC)	Early detection of synucleinopathy-related dementia
	Genetic variants	*GBA* , *SNCA* , and *LRRK2* polymorphisms associated with increased risk	Potential for personalized risk stratification
**Therapeutic implications**	Current treatments	Symptomatic management (cholinesterase inhibitors), non-pharmacological approaches	Limited disease-modifying therapies; early diagnosis critical
	Challenges	Late diagnosis, heterogeneity in progression, comorbidities, polypharmacy	Necessitates multidisciplinary care and future research

**Abbreviations:** CSF, cerebrospinal fluid; DTI, diffusion-tensor imaging; FDG-PET, fluorodeoxyglucose positron-emission tomography; *GBA* , *glucocerebrosidase, β, acid* ; *LRRK2* , *leucine-rich repeat kinase 2* ; MRI, magnetic resonance imaging; RBD, rapid eye movement sleep behavior disorder; REM, rapid eye movement; RT-QuIC, real-time quaking-induced conversion; *SNCA* , *synuclein alpha* ; SPECT, single-photon emission computed tomography.

## Therapeutic Challenges

The management of cognitive impairment associated with RBD presents numerous therapeutic challenges rooted in the complexity of its underlying neurodegenerative pathology, the prodromal nature of the disorder, and the current lack of disease-modifying treatments. Addressing these challenges is critical to optimize patient care and guide future research ( Table 2 ). 28 29 30

1. **Early Detection and Treatment Initiation**
One of the foremost challenges lies in the timely identification of cognitive decline in patients with iRBD. The cognitive deficits in iRBD are often subtle, non-specific, and may precede motor symptoms by years, making early diagnosis difficult. Without standardized screening protocols and widely-accepted biomarkers, many patients are diagnosed at a stage when neurodegeneration is already advanced, limiting the therapeutic window for intervention. 29 31 32 The absence of definitive clinical guidelines to systematically evaluate cognition in RBD further complicates early recognition and intervention. 31 33 34
2. **Limited Disease-Modifying Therapies**
Currently, no therapies have been conclusively shown to alter the natural progression of synucleinopathy-associated cognitive decline in RBD. Most pharmacologic interventions are symptomatic, focusing on alleviating cognitive symptoms or sleep disturbances rather than halting neurodegeneration. 21 23 24 Cholinesterase inhibitors, such as rivastigmine, have demonstrated some efficacy in improving cognitive symptoms in DLB and PDD, but evidence in the RBD population remains preliminary and anecdotal. The challenge remains to develop and validate novel neuroprotective agents that can be initiated during the prodromal phase to delay or prevent dementia onset. 13 15 17
3. **Managing Comorbidities and Polypharmacy**
Patients with RBD frequently exhibit a complex clinical profile, often including sleep fragmentation, mood disorders, autonomic dysfunction, and motor symptoms. These comorbidities complicate treatment strategies and may limit the tolerability and efficacy of medications used to address cognitive symptoms. 8 9 10 11 Furthermore, polypharmacy increases the risk of adverse effects, drug interactions, and non-adherence, particularly in older populations prone to cognitive decline. Tailoring individualized treatment plans that balance symptom control with safety remains an ongoing challenge. 16 18 19 20
4. **Variability in Cognitive Profiles and Progression Rates**
The heterogeneity in cognitive impairment observed among RBD patients—ranging from mild executive dysfunction to multidomain dementia—poses difficulties in prognostication and treatment selection. Some patients experience rapid progression, while others remain cognitively stable for years. 31 32 34 This variability complicates clinical trial design, as identifying suitable patient cohorts and appropriate endpoints requires nuanced understanding of disease trajectories. Personalized approaches to monitoring and treatment, informed by biomarkers and genetic profiling, are still in the early stages of development. 23 24 27
5. **Non-Pharmacological Interventions and Patient Engagement**
Non-pharmacological strategies, including cognitive rehabilitation, sleep hygiene optimization, and psychoeducation, offer potential benefits but remain underused and insufficiently studied in RBD-associated cognitive impairment. 31 33 34 Implementing such interventions requires multidisciplinary coordination and patient engagement, which may be hindered by the cognitive deficits themselves. Developing accessible and scalable programs tailored to this population represents an important therapeutic frontier. 33 35 36

**Table 2:** Therapeutic challenges

Therapeutic challenge	Description	Implications	Potential solutions/Future directions
**Early detection and treatment initiation**	Difficulty in identifying subtle cognitive decline in idiopathic RBD due to non-specific symptoms and lack of standardized screening and biomarkers	Late diagnosis reduces the therapeutic window, limiting early intervention opportunities	Development of validated biomarkers, standardized cognitive screening protocols, and clinical guidelines
**Limited disease-modifying therapies**	Absence of therapies that alter synucleinopathy progression; the current treatments are mainly symptomatic	Symptomatic management does not prevent or delay dementia onset, leaving an unmet need for neuroprotective agents	Research and development of novel neuroprotective drugs targeting the prodromal stages of RBD
**Managing comorbidities and polypharmacy**	Complex patient profiles with multiple symptoms and comorbidities; risks of drug interactions and adverse effects	Polypharmacy increases the risk of side effects and non-adherence, complicating treatment plans in vulnerable populations	Individualized treatment approaches balancing efficacy and safety; integrated multidisciplinary care
**Variability in cognitive profiles and progression rates**	Heterogeneous cognitive impairment ranging from mild to multidomain dementia; variable disease-progression rates	Challenges in prognosis, clinical trial design, and treatment personalization due to disease heterogeneity	Use of biomarkers, genetic profiling, and personalized monitoring strategies to tailor interventions
**Non-pharmacological interventions and patient engagement**	Underuse and limited research on cognitive rehabilitation, sleep hygiene, and psychoeducation in RBD	Lack of accessible programs and patient engagement hinders the holistic management of cognitive symptoms	Development of scalable, multidisciplinary, non-pharmacological programs tailored to RBD patients

**Abbreviation:** RBD, rapid eye movement sleep behavior disorder.

## Discussion

Rapid eye movement sleep behavior disorder has evolved from being considered a rare parasomnia to a clinically-significant prodromal marker of neurodegeneration, particularly within the spectrum of synucleinopathies. 7 8 9 The accumulating body of evidence 12 13 14 linking RBD to cognitive impairment underscores the complexity of the disorder and its diagnostic and prognostic relevance in contemporary neurology and sleep medicine.

The findings herein reviewed highlight several critical insights. First and foremost, cognitive deficits in RBD—especially in iRBD—are not incidental. Rather, they likely reflect an ongoing neurodegenerative process that affects brain regions far beyond the pontine structures traditionally implicated in REM sleep regulation. 31 32 34 The presence of cognitive dysfunction in patients with iRBD, particularly in domains such as attention, executive function, visuospatial processing, and episodic memory, suggests that neurodegeneration may already be active in cortical and subcortical networks well before the emergence of overt motor symptoms associated with disorders like PD or DLB. 24 26 27

### RBD as a Prodromal Neurodegenerative State

One of the most striking implications of the RBD-cognition link is the potential to detect neurodegenerative diseases at a prodromal stage—years or even decades before the classic symptoms arise. Longitudinal cohort studies 5 6 7 have shown that up to 80% of the individuals with iRBD may eventually develop a synucleinopathy, with a substantial proportion manifesting cognitive impairment as a primary or early feature. This timeline challenges traditional diagnostic paradigms and suggests that RBD could serve as a unique entry point for neuroprotective intervention, ideally before significant neuronal loss occurs. 18 19 20 22

Moreover, the pattern of cognitive deficits observed in iRBD patients shares a remarkable overlap with those observed in early DLB, including deficits in executive functioning, attentional control, and visuospatial reasoning. 33 35 36

Longitudinal studies and consensus statements 37 38 39 have established that iRBD often precedes neurodegenerative disorders such as PD and DLB, highlighting its role as a prodromal biomarker. This prodromal phase offers a window for early detection and potential intervention before overt motor or cognitive symptoms manifest. These similarities strengthen the hypothesis that iRBD may represent an early clinical manifestation of DLB or PDD, rather than a separate disease entity. As such, early recognition of cognitive changes in RBD could help predict disease trajectory and inform patient counseling, risk stratification, and research participation. 9 10 11

### Neurobiological Mechanisms: A Common Pathology

From a neuropathological perspective, alpha-synuclein aggregation remains the most consistent molecular hallmark connecting RBD to cognitive decline. 12 23 The progression of alpha-synuclein pathology is thought to follow a caudal-to-rostral trajectory, initially affecting the lower brainstem nuclei involved in REM atonia and later spreading to the limbic system, basal forebrain, and neocortical areas. 25 26 27 This cascade aligns with Braak's staging model of PD and supports the hypothesis that the same pathological process may underlie sleep-related motor behaviors and cognitive deterioration. 31 32 33 34

Recent evidence 40 suggests heterogeneity in the propagation of synuclein pathology, with some cases following a brain-first (top-down) route, while others follow a body-first (bottom-up) progression, impacting the onset and pattern of cognitive decline. In addition to alpha-synucleinopathy, cholinergic deficits appear to play a central role. Cholinergic pathways, particularly those originating in the pedunculopontine nucleus and the basal forebrain, are critically involved in REM sleep modulation and higher-order cognitive processing. 7 8 9 Dysfunction in these systems likely contributes to the attentional and executive deficits commonly reported in iRBD, and it may partially explain the efficacy of cholinesterase inhibitors in improving cognitive symptoms in some patients with RBD-associated neurodegeneration. 12 13 14 26

### Neuroimaging and Biomarkers: Mapping the Cognitive Decline

Neuroimaging has emerged as an essential tool in delineating the neural substrates of cognitive impairment in RBD. Structural MRI findings in iRBD patients have demonstrated cortical thinning in the frontal, parietal, and temporal lobes—regions associated with executive control and memory encoding. 33 35 36 Functional imaging techniques, including FDG-PET and SPECT, further corroborate these findings by revealing hypometabolism in posterior cortical areas, particularly the occipital lobe—an imaging pattern characteristic of early DLB. These converging data support the notion that neurodegeneration is already widespread by the time RBD becomes clinically apparent. 5 7 8 9

Furthermore, the application of molecular and fluid biomarkers has added a new dimension to our understanding. Decreased CSF amyloid-beta, increased phosphorylated tau, and positive alpha-synuclein seeding assays (such as RT-QuIC) in iRBD patients who later convert to DLB or PDD provide compelling evidence of shared pathological mechanisms. 16 18 19 20 These biomarkers, while still under development, hold great promise for early differential diagnosis and therapeutic targeting. 18 20

Recent studies from the Montreal group have provided valuable insights into cognitive decline in iRBD. Haddad et al. 41 (2025) have demonstrated that increased free water in specific brain regions predicts conversion to DLB. Rahayel et al. 42 (2024) have identified 99m technetium-hexamethyl-propylenamine oxime (99mTc-HMPAO) SPECT perfusion signatures associated with clinical progression in iRBD. And Joza et al. 43 (2025) have reported distinct subtypes of brain atrophy progression underlying phenoconversion. Together, these findings emphasize the heterogeneity in neurodegenerative trajectories and underscore the importance of advanced imaging biomarkers in prognostication.

### Clinical Implications and Future Directions

From a clinical standpoint, the recognition of cognitive impairment in RBD is of utmost importance. Early neuropsychological assessment should be an integral component of the diagnostic workflow in patients with suspected or confirmed RBD. Regular cognitive monitoring may help identify individuals at the highest risk of progression and enable timely referral to neurology or memory clinics.

Although treatment options are currently limited, preliminary evidence suggests that cholinesterase inhibitors (such as rivastigmine) may alleviate cognitive symptoms in RBD patients, particularly those progressing toward DLB. Cognitive rehabilitation, sleep hygiene strategies, and tailored psychoeducation may also enhance functional outcomes. However, no disease-modifying therapies are yet available, and the development of such treatments remains a critical research priority.

Emerging genetic data further suggest that certain polymorphisms—such as those in *GBA* and *SNCA* —may predispose individuals to RBD and cognitive decline, offering potential avenues for personalized medicine. Incorporating genetic screening into risk-assessment models may enhance predictive accuracy, especially when combined with biomarker and imaging data.

## Limitations and Research Gaps

Despite these advances, significant knowledge gaps remain. The exact mechanisms by which RBD leads to cognitive dysfunction are not yet fully understood. Furthermore, most studies to date have been observational and heterogeneous in methodology, limiting the generalizability of the findings. Standardized diagnostic criteria for cognitive impairment in RBD, harmonized across centers and longitudinal designs, are needed to facilitate large-scale clinical trials.

Moreover, while many studies have focused on patients with iRBD, less is known about the trajectory of cognitive impairment in secondary RBD, such as that arising in the context of established PD or MSA. Whether therapeutic interventions targeting REM sleep disturbances can slow cognitive decline also remains an open question worthy of exploration. Bridging these gaps necessitates international collaboration, harmonization of methodologies, and incorporation of emerging biomarkers to enable early-phase trials of disease-modifying therapies.

## Conclusion

In summary, RBD is far more than a parasomnia; it is increasingly recognized as a prodromal manifestation of neurodegenerative disease, often heralding the onset of cognitive impairment and dementia. The shared neuropathological underpinnings between RBD and cognitive decline—particularly involving alpha-synuclein accumulation and cholinergic dysfunction—underscore the importance of early identification and longitudinal cognitive monitoring in affected individuals.

As research continues to elucidate the biological mechanisms, diagnostic markers, and therapeutic targets linking RBD and cognitive dysfunction, clinicians must remain vigilant. Timely recognition and proactive management of cognitive symptoms in RBD may not only enhance patient outcomes but also offer a unique window of opportunity for intervention at a stage when neurodegeneration may still be modifiable.

## Key Findings

1. **Early Cognitive Impairment in RBD** :
  Individuals with iRBD frequently show subtle deficits in executive function, attention, visuospatial abilities, and memory, often preceding the motor symptoms of PD or dementia by years.
2. **Pathophysiological Mechanisms** :
  - Alpha-synuclein aggregation spreads from REM-regulating brainstem nuclei to limbic and neocortical regions, paralleling cognitive decline.
  - Cholinergic system degeneration (pedunculopontine nucleus, basal forebrain) contributes to REM atonia loss and cognitive deficits.
  - Frontostriatal and limbic circuit involvement explains executive, memory, and emotional regulation impairments.
3. **Neuroimaging Correlates** :
  - MRI shows cortical thinning in frontal, temporal, and parietal lobes, plus hippocampal atrophy.
  - FDG-PET and SPECT reveal posterior cortical hypometabolism.
  - Diffusion-tensor imaging (DTI) indicates disrupted frontostriatal connectivity.
4. **Biomarkers** :
  - CSF alterations: decreased amyloid-beta, increased tau, positive alpha-synuclein seeding assays (RT-QuIC).
  - Genetic variants ( *GBA* , *SNCA* , *LRRK2* ) increase the risk and may influence progression.
5. **Therapeutic Challenges** :
  - Early detection is difficult due to subtle cognitive changes and lack of standardized screening.
  - There are no disease-modifying treatments; the current management is primarily symptomatic.
  - Comorbidities, polypharmacy, and heterogeneous cognitive profiles complicate care.
  - Non-pharmacological interventions remain underutilized but offer potential benefits.
6. **Clinical Implication** :
  Rapid eye movement sleep behavior disorder serves as a robust prodromal biomarker for synucleinopathy-related cognitive decline. Early identification using neuropsychological testing, imaging, and molecular diagnostics is crucial for monitoring and for a potential intervention.
